# Perspectives and awareness of environmental sustainability in the infection prevention and control community nationally

**DOI:** 10.1017/ash.2024.439

**Published:** 2024-10-07

**Authors:** Abarna Pearl, Dana E. Pepe, Preeti Mehrotra

**Affiliations:** 1 Division of Infectious Diseases, Beth Israel Deaconess Medical Center, Boston, MA, USA; 2 Division of Infection Control/Hospital Epidemiology, Beth Israel Deaconess Medical Center, Boston, MA, USA

## Abstract

In this survey of infection prevention and control (IPC) professionals, we gauged knowledge, attitudes and institutional practices related to environmental sustainability and IPC. Overall, IPC professionals have not yet universally adopted measures to promote environmental sustainability. More research is needed around environmentally sustainable efforts that preserve patient safety in IPC.

## Background

In the United States (US), the healthcare sector generates 6 million tons of waste annually, and accounts for 8.5% of national carbon emissions.^1,2^ Healthcare’s environmental impact became especially apparent during the COVID-19 pandemic, when single-use plastic consumption skyrocketed due to greater demand for personal protective equipment (PPE).^3^ Byproducts of plastic degradation include methane, a potent greenhouse gas, and may harm human health.^4,5^ Yet there is minimal recognition of this issue, particularly within decision-making in the field of Infection Prevention and Control (IPC). The aim of our study was to gauge general knowledge and attitudes of hospital epidemiologists (HEs) and infection preventionists (IPs) around the intersection of environmental sustainability and IPC, as well as to identify related institutional practices.

## Methods

An online survey composed of ten questions related to environmental sustainability in IPC was emailed to members of the SHEA Research Network (SRN), a national consortium of healthcare facilities collaborating on IPC research, from August–October 2023. Three questions concerned demographics, two addressed knowledge, two addressed attitudes and there were three regarding institutional practices. All questions were multiple choice barring one which required a free-form text answer. Survey answers were collated via Redcap^©^ and descriptive results were obtained. See supplementary material for full survey.

## Results

Forty-two individuals (33 HEs, 7 IPC Directors, and 2 IPs) from unique institutions completed the survey, resulting in a response rate of 45%. There were no incomplete responses. Thirty (71.4%) were from academic medical centers, 5 (11.9%) were from VA medical centers and 7 (16.7%) were from community hospitals. Six (14.3%) participants were from institutions with >1000 beds, 15 (35.7%) from institutions with 500–1000 beds, 19 (45.2%) from institutions with 100–500 beds and 2 (4.8%) from institutions with <100 beds.

Over half of participants correctly estimated the amount of waste and carbon emissions produced annually by the US healthcare system. Conversely, 42.9% considered environmental sustainability concerns either important or very important in IPC decision-making. The majority (47.6%) considered the issue moderately important, 9.5% slightly important and none considered it unimportant.

**Figure 1.**
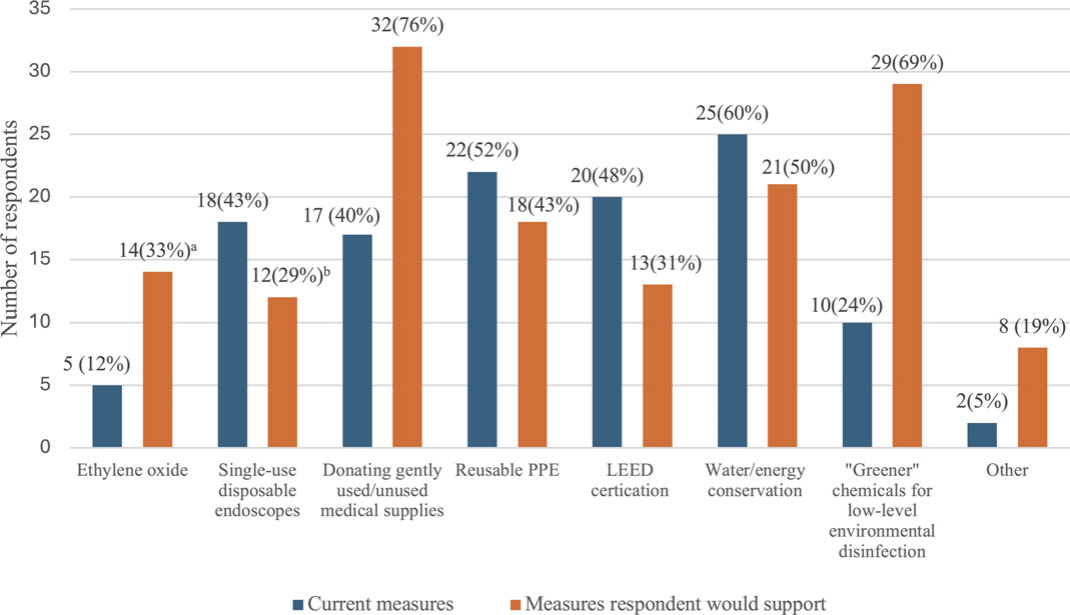
Current institutional practices, and policies respondents would support in the future, related to sustainability in infection prevention and control. ^a^Respondent would support elimination of ethylene oxide use. ^b^Respondent would support elimination of single-use, disposable endoscopes.

Fifteen (35.7%) respondents had an environmental sustainability committee at their institution and of these, 8 had an established relationship between the committee and the IPC department. The most common techniques to promote sustainability amongst institutions were water/energy conservation (59.5%), reusable PPE (52.4%) and Leadership in Energy and Environmental Design (LEED) certification (47.6%) (Figure 1). Highlighting the current challenges in incorporating sustainability in IPC, 18 (42.9%) reported use of single-use disposable flexible scopes (ie, endoscopes, bronchoscopes), 17 (40.5%) reported donating gently used, expired or unused medical supplies, and 10 (23.8%) reported use of “greener” chemicals for low-level environmental disinfection, while 5 (11.9%) still reported use of ethylene oxide (ETO) as part of sterilization efforts.

When asked which efforts they would support at their institutions to promote environmental sustainability, 28.6% of participants would eliminate the use of single-use endoscopes and instead rely on high level disinfection/sterilization and one third would avoid use of ETO for sterilization (Figure 1). Thirty-two respondents (76.2%) would promote donation of gently used, expired or unused medical supplies, 69% would use “greener” chemicals for low-level environmental disinfection, 50% would implement water conservation (ie, low flow aerators) or energy conservation measures (ie, motion sense lighting), 42.9% would purchase reusable PPE and 31.0% would pursue LEED certification. Other suggested mechanisms included optimizing use of PPE for transmission-based precautions, including discontinuing contact precautions for methicillin-resistant *Staphylococcus aureus* (MRSA) and/or vancomycin-resistant *Enterococcus* (VRE), management of single-use items for patients on contact precautions, moving to virtual meetings to reduce travel-related emissions, and recycling food matter from the cafeteria.

In deciding whether to support environmental sustainability measures, key considerations participants articulated were patient safety concerns, knowledge about effectiveness and costs, and garnering administrative support, as well as buy-in from other departments. A salient theme amongst responses was the concern for transmission of infections, including multi-drug resistant organisms (MDROs), associated with re-using instruments, in particular, endoscopes. Other factors included impacts on work flow/personnel time, Instructions For Use (IFU) by manufacturers supporting use of agents other than ETO, regulatory risk and liability associated with reusable devices and PPE, infrastructure (eg, for reusable gowns), resources for distributing gently used, expired or unused items, and issues related to ageing physical plants. Selected illustrative free-text responses are displayed in Table 1. See supplementary tables S1 – S3 for all free text answers.

**Table 1.** Selected participant quotes on key considerations in supporting environmentally sustainable measures in infection prevention and control

**Quotes regarding the balance between patient safety and sustainability**
In general, trade-offs between patient safety and environmental impact has weighed heavily to the patient safety side and waste/environmental impact has not been considered as an important variable.
IP [infection prevention] and sustainability often come with tradeoffs, and patient safety can and should trump sustainability concerns.
“Greener” chemicals might be interesting, but we are trying to keep the wheels on the bus and prevent infections. Environmental sustainability is more of an afterthought.
Our focus is on understanding--and appropriately weighing--all the factors that go into product or energy usage. For example, when considering disposable endoscopes, consider the differential value (compared to re-processed) for: clinical functionality, cost (device and reprocessing), waste, contamination risk, subsequent patient infection risk. While the environmental perspective may favor zero use of disposables, and the infection prevention perspective may favor 100% use of disposables to make transmission risk zero, the holistic “best” answer is probably very limited use in carefully selected situations.
**Quotes regarding regulatory risk**
A key concern is that reusable equipment could increase the chance of transmitting infection in the healthcare setting. Any location where high-level disinfection or sterilization is done is a regulatory liability because HLD [high-level disinfection] and sterilization processes are frequently cited by regulatory and accreditation organizations and create regulatory risk for the hospital. When single use equipment is available, it eliminates that risk.
Many regulations prevent reuse without extensive proof that we will not have a minimal increment in risk. There are few voices to advocate for a reasonable middle ground.
**Quotes regarding the need for more guidance by manufacturers and regulatory bodies**
Some items are very difficult to clean. Cleaning takes manpower and time both of which are in low supply right now. Manufacturers have not standardized to a narrow selection of products, so we are struggling to stay within MIFUs [manufacturer’s instructions for use] and use a small range of products to clean with…Organisms are getting more difficult to eradicate so use of disposable is more favorable for some patients with high consequence organisms.
Per the manufacturer IFU [instructions for use], certain types of equipment can only be sterilized using ETO [ethylene oxide]. We had tried to eliminate it but had to re-institute it for this reason. We need manufacturers to come up with other validated ways to sterilize equipment.
**Quotes regarding other challenges with implementing environmentally sustainable procedures**
I expect pushbacks from frontline for cost, fear of safety related to reusable PPE [personal protective equipment], and questions “why we need to change the practice.”
Our gowns contain PFAs [perfluoroalkoxy alkanes] so we are weighing the flushing of PFAs down the drain versus the use of disposable gowns. We cannot seem to find reusable gowns without PFAs.
Water conversation measures may increase risk of *Legionella.*

## Discussion

Although there is growing awareness around the contribution of health care to greenhouse gas emissions and waste production, IPC professionals have yet to universally adopt measures that promote environmental sustainability. In our survey, most participants did not prioritize environmental sustainability when making decisions regarding IPC. Given the central role of IPC teams in regulating the use of environmentally active chemicals, water management and PPE in health care, this is a missed opportunity.

Notably, 8 out of 42 (19.0%) participants reported an established relationship between the institution’s environmental sustainability committee and IPC. This is a clear and actionable area for improvement. The establishment of environmental sustainability committees universally in healthcare entities, and greater discourse between these and IPC departments, may facilitate durable steps to reducing individual institutions’ environmental footprints. Additionally, ending use of ETO, a known carcinogen, for device sterilization is an uncontested move, which has been championed by the Environmental Protection Agency.^6^ Another potentially high-yield measure suggested by participants includes discontinuing use of PPE in settings where its utility for transmission-based precautions has been challenged in the literature.^7^

Many participants acknowledged the dilemma of balancing patient safety and sustainability matters. A frequent concern was that reusable equipment, eg, PPE and endoscopes, may lead to transmission of infectious pathogens, likely in the wake of multiple recent outbreaks of MDRO infections associated with reusable duodenoscopes.^8^ Manufacturers and regulatory bodies should be included in multi-disciplinary conversations that weigh the difficulty of reprocessing, gauge regulatory risk, and yet also promote environmental sustainability to create “middle ground” approaches.

Furthermore, research, eg, life cycle assessments ascertaining the environmental impact of each item or process, should be undertaken to empirically determine the most sustainable policies.^9,10^ For instance, while reusable endoscopes may generate less plastic waste than their disposable counterparts, we must also consider the environmental impact of the supply chain, and chemical byproducts, associated with using and re-processing these products. This is particularly important as the production and transportation of goods and services used by the health sector is thought to comprise 80% of healthcare’s carbon footprint^2^. Evidence-based recommendations may lead to greater confidence in promoting sustainable policies within the IPC community. However, it is telling that less than half of participants reported LEED certification, a globally recognized green building rating system, which may support IPC and sustainability goals using advanced building design strategies that improve indoor air quality, water efficiency, optimize ventilation, and reduce pathogen transmission.

To our knowledge, this is the first study to gauge attitudes, knowledge and practices of IPC professionals in the US regarding environmental sustainability. Another strength is the insights gained from free text answers. Limitations include the low response rate.

Our study demonstrates the need for more research and education to inform decisions around environmentally sustainable efforts in IPC that also preserve patient safety. Professional and regulatory bodies must acknowledge and promote the importance of environmental sustainability in IPC decision-making moving forward.

## Supporting information

Pearl et al. supplementary material 1 Pearl et al. supplementary material

Pearl et al. supplementary material 2 Pearl et al. supplementary material

## Supplementary material

The supplementary material for this article can be found at https://doi.org/10.1017/ash.2024.439.
