# Supplementary material for: Perspectives and awareness of environmental sustainability in the infection prevention and control community nationally
Source: Antimicrob Steward Healthc Epidemiol. 2024 Oct 7;4(1):e165. doi: 10.1017/ash.2024.439 (PMC11474868; doi:10.1017/ash.2024.439)
Supplement: Pearl et al. supplementary material 1 — Pearl et al. supplementary material [file S2732494X2400439Xsup001.docx]

**Perspectives and Awareness of Environmental Sustainability in the Infection Prevention and Control Community Nationally – Survey Questions.**

**Background:**

Healthcare contributes significantly to waste production and greenhouse gas emissions. This is especially true in the aftermath of the ongoing COVID-19 pandemic. Yet there is little recognition of this growing issue in healthcare, particularly as it relates to the field of infection prevention. As a result, environmental sustainability is typically not routinely considered in infection prevention decision-making. Moreover, in some situations it remains unclear which approach would minimize environmental impact.

The Environmental Protection Agency defines environmental sustainability as "creating and maintaining the conditions under which humans and nature can exist in productive harmony to support present and future generations." In reference to health care, this involves balancing patient outcomes with economic, environmental, and social costs, and could include reducing carbon emissions, and minimizing biohazardous and/or non-biodegradable waste.

**Description of study:** This survey is designed to gauge knowledge and attitudes of hospital epidemiologists and infection preventionists around the intersection of environmental sustainability and infection prevention. We also aim to identify current institutional practices regarding environmental sustainability within Infection Prevention and Control (IPC). Survey participants should be familiar with healthcare epidemiology workflows and policies within their institutions, and may consult other members of their IPC team in responding to the survey.

Please complete the survey below. Thank you!

1. What is your SHEA research network institution number?
2. What is your role within infection prevention?

a) Hospital epidemiologist

b) Director of infection prevention

c) Infection control practitioner

1. How would you best describe your institution?

a) Academic medical center

b) Veterans Affairs (VA) medical center

c) Community hospital

1. How many beds does your institution have?

a) <100

b) 100-500

c) 500-1000

d) >1000

1. If you had to guess, how much total waste is generated by the United States (US) healthcare industry annually?

a) 1-2 million tons

b) 2-3 million tons

c) 3-4 million tons

d) 5-6 million tons

1. If you had to guess, what proportion of total carbon emissions in the US is generated by the healthcare industry annually?

a) 1-5 %

b) 5-10%

c) 10-20%

d) >20%

1. How important do you think environmental sustainability concerns are when making decisions about infection control?

a) Very important

b) Important

c) Moderately important

d) Slightly important

e) Unimportant

1. Do you have an environmental sustainability committee in your institution

a) Yes

b) No

c) Don’t know

- 1. If yes, does your infection control department have a relationship with the committee?
     1. Yes
     2. No
     3. Don’t know

1. Which of the following processes apply to your institution? Check all which are applicable.

- Use of single-use disposable flexible scopes (i.e., endoscopes, bronchoscopes)
- Use of ethylene oxide as part of sterilization efforts
- Donating gently used, expired or unused medical supplies
- Reusable personal protective equipment (PPE) (i.e., gowns, respirators)
- LEED certification
- Water conservation (i.e. low flow aerators) or energy conservation measures (motion sense lighting)
- Use of “greener” chemicals for low-level environmental disinfection
- Other

If other, please specify: _________________________________________________

1. In your role within infection prevention, which of the following efforts would you support at your institution to promote environmental sustainability? Check all that apply.

- Eliminate use of single-use disposable flexible scopes (i.e., endoscopes, bronchoscopes) and rely on high level disinfection/sterilization
- Eliminate use of ethylene oxide as part of sterilization efforts
- Promote donation of gently used, expired or unused medical supplies
- Purchase reusable personal protective equipment (PPE) (i.e., gowns, respirators)
- Pursue LEED certification
- Implement water conservation (i.e. low flow aerators) or energy conservation measures (motion sense lighting)
- Use of “greener” chemicals for low-level environmental disinfection
- Other

If other, please specify: _________________________________________________

1. What would be your key considerations, including potential barriers, in deciding whether or not to support one or more of the above efforts in your institution? Please explain.
