# Supplementary material for: Perspectives and awareness of environmental sustainability in the infection prevention and control community nationally
Source: Antimicrob Steward Healthc Epidemiol. 2024 Oct 7;4(1):e165. doi: 10.1017/ash.2024.439 (PMC11474868; doi:10.1017/ash.2024.439)
Supplement: Pearl et al. supplementary material 2 — Pearl et al. supplementary material [file S2732494X2400439Xsup002.docx]

**Supplementary table S1. “Which of the following processes apply to your institution?” Free-text responses to the “other” category**

| Plethora of projects related to decreasing OR [operating room] waste; recently discontinued ETO [ethylene oxide] use |
| --- |

**Supplementary table S2. “In your role within infection prevention, which of the following efforts would you support at your institution to promote environmental sustainability?” Free-text responses to the “other” category**

| Optimize use of PPE [personal protective equipment] for TBP [transmission-based precautions] based on available data, specifically MDROs [multi drug resistant organisms] and duration of precautions |
| --- |
| Limit disposable gown use |
| 1. Review reprocessing that does not lead to infection transmission; 2. more consideration for types of infections/colonization or clinical syndromes demonstrating impact with gown/glove use (example: remove isolation for MRSA [*methicillin-resistant Staphylococcus aureus*] colonization; 3. limit isolation for VRE [*vancomycin-resistant Enterococcus*]); 4. assessing single use items and how these are managed for patients on contact isolation |
| Consider discontinuing practices that consume resources but are demonstrably beneficial (e.g., selected or complete discontinuation of contact precautions for MRSA and/or other pathogens) |
| Decreasing the use of single use vials in situations where multiuse vials may be safe and effective (e.g. single use vials of eye drop medications) |
| I'm not sure what can be done to be greener.  Moving to Teams/Zoom meetings as an option for most all meetings has reduced travel, gas and increased productivity. |
| We recycle all food stuffs from the cafeteria |

**Supplementary table S3. Complete table of quotes for survey question, “What would be your key considerations, including potential barriers, in deciding whether or not to support one or more of the above efforts in your institution? Please explain.”**

| Balancing measures against infection prevention policy, adherence metrics and outcome metrics. |
| --- |
| Costs are significant barriers to many of the efforts we suggest for environmental conservation. |
| Cost and impact to workflows/staffing needs would be primary concerns |
| In general, trade-offs between patient safety (single use, disposable, etc) and environmental impact (contribution to landfill, wasted product) has weighed heavily to the patient safety side and waste/environmental impact has not been considered as an important variable. |
| Several concerns:  1. Per the manufacturer IFU [instructions for use], certain types of equipment can only be sterilized using ETO [ethylene oxide]. We had tried to eliminate it but had to re-institute it for this reason. We need manufacturers to come up with other validated ways to sterilize equipment.  2. Some water conservation measures can actually increase risk of waterborne infections (low flow --> more biofilm, etc.). There needs to be more focus on this balance.  3. We are struggling right now with green cleaners vs. disinfectants for use on the floors of patient rooms. For multiple reasons (damage, stickiness, etc.) our ESD [environmental services] and Safety teams do not support disinfectants on floors, even though we're concerned about persistence of microorganisms and spores on floors in patient areas. This has been a real challenge. |
| IP [infection prevention] and sustainability often come with tradeoffs, and patient safety can and should trump sustainability concerns. |
| Some items are very difficult to clean.  Cleaning takes manpower and time, both of which are in low supply right now. Manufacturers have not standardized to a narrow selection of products, so we are struggling to stay within MIFUs [manufacturer’s instructions for use] and use a small range of products to clean with.  Right now, it is out of control.  Organisms are getting more difficult to eradicate, so use of disposable is more favorable for some patients with high consequence organisms. |
| Expense, opportunity costs or competing priorities for management time, networking resources e.g. users of expired or unused medical supplies |
| Effectiveness |
| A key concern is that reusable equipment could increase the chance of transmitting infection in the healthcare setting.  Any location where high-level disinfection or sterilization is done is a regulatory liability because HLD [high-level disinfection] and sterilization processes are frequently cited by regulatory and accreditation organizations and create regulatory risk for the hospital.  When single use equipment is available, it eliminates that risk. |
| Lack of data on impact that reusable PPE [personal protective equipment] would have on transmission of pathogens. Water conversation measures may increase risk of Legionella (https://www.frontiersin.org/journals/water/articles/10.3389/frwa.2022.966223/full) |
| For many of these, which scenarios allow for more clinical efficiency, for cost savings, and for improving ease of practice without compromising patient safety. |
| Financial challenges; balancing impact on reducing healthcare associated infections. |
| Need proven efficacy with alternative strategies |
| Cost of implementation of changes |
| Our focus is on understanding--and appropriately weighing--all the factors that go into product or energy usage.  For example, when considering disposable endoscopes, consider the differential value (compared to reprocessed) for: clinical functionality, cost (device and reprocessing), waste, contamination risk, subsequent patient infection risk. While the environmental perspective may favor zero use of disposables, and the infection prevention perspective may favor 100% use of disposables to make transmission risk zero, the holistic "best" answer is probably very limited use in carefully selected situations. |
| Disposable supplies are better to prevent the spread of disease to other patients and staff. Water aerators cause bacterial buildup. Sterilization and disinfection guidelines. |
| Challenge with HLD for scopes is the issue with outbreaks, but would be in favor of reusable/washable gowns |
| Cost and effectiveness |
| Cost and effectiveness |
| Cost |
| I expect pushbacks from frontline for cost, fear of safety related to reusable PPE, and questions "why we need to change the practice". |
| Elimination of disposable ERCP [endoscopic retrograde cholangiopancreatography] scopes would put patients at risk of MDRO [multi drug resistant organism] infections due to the inability to adequately disinfect reusable scopes |
| Supply chain structure. Importance of preventing spread of important organisms – CRE [carbapenem-resistant *Enterobacterales*], *Candida auris.* |
| Administrative support |
| Costs; support from administration; impact on personnel time/effort; impact on workflow |
| Infrastructure for re-usable gowns |
| "Greener" chemicals might be interesting, but we are trying to keep the wheels on the bus and prevent infections.  Environmental sustainability is more of an afterthought.  Drugs/antibiotics entering the wastewater stream is a higher priority.  We moved from disposable PPE to a heavy investment into reusable gowns, HALO respirators (have about 1000) after COVID curtailed availability. |
| Effectiveness |
| Sustainability and IP&C [Infection Prevention and Control] leaders are already co-engaged to ensure the organization is giving due consideration to environmental impact and potential infection-/transmission-related harm for major initiatives. Aged facilities is a challenge for both IP&C and Sustainability. |
| Many regulations prevent reuse without extensive proof that we will not have a minimal increment in risk. There are few voices to advocate for a reasonable middle ground. |
| Buy in from other departments.  Need for leadership to direct departments to change. E.g. sterile supply, engineering etc. |
| Our gowns contain PFAs [perfluoroalkoxy alkanes] so we are weighing the flushing of PFAs down the drain versus the use of disposable gowns.  We cannot seem to find reusable gowns without PFAs. |
